# Predictors and Assessment of Hospice Use for End-Stage Renal Disease Patients in Taiwan

**DOI:** 10.3390/ijerph19010085

**Published:** 2021-12-22

**Authors:** Hung-Cheng Chen, Chien-Yi Wu, Hui-Ya Hsieh, Jiun-Shiuan He, Shang-Jyh Hwang, Hui-Min Hsieh

**Affiliations:** 1 Department of Nursing, Kaohsiung Medical University Hospital, Kaohsiung Medical University, Kaohsiung 80708, Taiwan; olive575589@gmail.com (H.-C.C.); 820065@kmuh.org.tw (H.-Y.H.); 2 Department of Family Medicine, Kaohsiung Medical University Hospital, Kaohsiung Medical University, Kaohsiung 80708, Taiwan; dietcokewu0822@gmail.com; 3 Department of Medical Research, Kaohsiung Medical University Hospital, Kaohsiung Medical University, Kaohsiung 80708, Taiwan; heshen0704@gmail.com; 4 Division of Nephrology, Department of Internal Medicine, Kaohsiung Medical University Hospital, Kaohsiung Medical University, Kaohsiung 80708, Taiwan; sjhwang@kmu.edu.tw; 5 Department of Public Health, Kaohsiung Medical University, Kaohsiung 80708, Taiwan; 6 Department of Community Medicine, Kaohsiung Medical University Hospital, Kaohsiung Medical University, Kaohsiung 80708, Taiwan; 7 Center for Big Data Research, Kaohsiung Medical University, Kaohsiung 80708, Taiwan; 8 Research Center for Environmental Medicine, Kaohsiung Medical University, Kaohsiung 80708, Taiwan

**Keywords:** end-stage renal disease, hospice care, palliative care, advance care planning, Patient Right to Autonomy Act

## Abstract

Objectives: Hospice and early palliative care are generally considered as an alternative and supportive care to offer symptoms relief and optimize the quality of life among end-stage renal disease (ESRD) patients, but hospice care remains underutilized. This study aimed to examine patient and health system characteristics and develop a patient assessment scale to evaluate ESRD patients for hospice care after the implementation of non-cancer hospice care reimbursement policy in 2009 in Taiwan. Method: We conducted a retrospective cohort study using nationwide population-based datasets. Adult long-term dialysis patients between 2009 and 2012 were included. Multivariable logistic regression and the Firth penalized likelihood estimation were used to estimate the likelihood of receiving hospice care. A receiver operating characteristic curve (ROC) analysis and C-statistic were calculated to determine the optimal models for a patient assessment of hospice use. Results: Patients who were older, comorbid with anemia (odds ratio [OR] 3.53, 95% CI 1.43–8.70) or sepsis (OR 1.62, 95% CI 1.08–2.44), with longer dialysis durations, more hospitalizations (OR 4.68, 95% CI 2.56–8.55), or primary provider care with hospice (OR 5.15, 95% CI 2.80–9.45) were more likely to receive hospice care. The total score of the patient assessment scale of hospice care was 0–28 with a cut-off value of 19 based on the results of the receiver operating characteristic curve. Conclusion: Given the “Patient Right to Autonomy Act” implemented in Taiwan in 2019 to promote the concept of a “good quality of death”, this patient assessment scale may help health professionals target ESRD patients for hospice care and engage in shared decision making and the advance care planning process.

## 1. Introduction

End-stage renal disease (ESRD) is a major cause of morbidity and mortality worldwide due to aging populations and the increasing prevalence of chronic kidney disease (CKD) [1]. The number of patients with ESRD receiving renal replacement therapy (RRT) is projected to increase from 2618 million in 2010 to 5439 million worldwide by 2030 [1]. In Taiwan, the prevalence of CKD is high, and ESRD incidence and prevalence are the highest in the world, based on United States Renal Data System international comparisons. The national prevalence of CKD was approximately 11.9%, with a total burden of more than 2.5 million people in Taiwan, and the proportion of ESRD patients receiving hemodialysis and peritoneal dialysis was 90.6% and 9.4% in 2018, respectively [2,3]. In Taiwan, the average age of ESRD patients initiating dialysis in 2018 was 68.4 years for hemodialysis and 57.1 years for peritoneal dialysis. Approximately 61.2% of patients older than 65 years had initiated dialysis and 48.9% of prevalent cases had endured dialysis for more than 5 years [3]. Patients receiving long-term continuous dialysis may have a lower quality of life, increased dependence on others, and severe symptoms (i.e., pain, nausea, insomnia, anxiety, or depression) and other physical and functional deficits [4].

Hospice and early palliative care are generally considered as an alternative and supportive care to offer symptoms relief and optimize the quality of life among ESRD patients [2,3,4,5,6,7]. However, discontinuing intensive dialysis and receiving hospice care can be a more difficult decision than withholding treatment, and thus, hospice remains underutilized for ESRD patients [8]. Previous studies suggested that patients who decided to forgo dialysis were most likely older with poor functional status, but most evidence was from western countries [9,10]. For example, Chen et al. (2018) investigated factors associated with withdrawal from hemodialysis before death in a single tertiary center in the United States from 2001 to 2013, and found acute medical complications, or failure to thrive/frailty were major reasons for withdrawal [11]. Terminally ill patients may often lack the decision-making capacity and autonomy to terminate unwanted medical interventions that prolong the dying process [12]. Advance care planning became important for patients with ESRD, particularly those patients that do not have decision-making capacity near the end-of-life and are unable to express care preferences [5,8,13]. In addition to patient clinical functional factors, other potential reasons may also affect the decision to receive hospice care, such as cross-cultural and social customs, patient- and family-related factors (e.g., inconsistency in family values, lack of information about hospice), physician-related factors (e.g., lack of understanding of the concept of withdrawing or uncertainty about implementation), or health system-related issues (e.g., lack of availability of community-based hospice care systems) [14]. Given cross-cultural differences relating to dialysis withdrawal and hospice care, empirical studies are necessary to address factors associated with the preference for hospice care in ESRD patients undergoing long-term dialysis in Asian countries, particularly Taiwan, which has a high incidence and prevalence of ESRD. 

In Taiwan, a non-cancer hospice care reimbursement policy for ESRD patients was implemented nationwide by the National Health Insurance Administration (NHIA) in 2009 in an effort to provide inpatient hospice care to terminally ill patients who do not have cancer, including ESRD patients [14,15]. Before the policy reform, there were several policy reforms in palliative care for different patient groups in Taiwan. First, the Statute for Palliative Care, June 2000, was an official law promoting palliative care, which provides terminally ill patients the right to receive hospice care and to issue do-not-resuscitate orders by their free will. Taiwan was the first country in Asia to legislate this official law affirming the right of terminally ill patients [14]. In July 2000, the NHIA initiated a per diem payment for hospitals to provide hospice care only for terminal cancer patients. Approximately 66 hospitals throughout Taiwan provide inpatient hospice care, receiving per diem payments. After September 2009, the NHIA also began reimbursing hospitals for the provision of hospice care to non-cancer terminally ill patients, including those with acute or chronic renal failure [14,16,17]. Criteria for enrolling ESRD patients in hospice care include: (1) advanced CKD (stage 4, 5, or ESRD) with or without RRT (hemodialysis, peritoneal dialysis, kidney transplant); (2) expectation of death due to severe uremia symptoms; (3) unwillingness to undergo long-term dialysis or kidney transplant under clear consciousness; or (4) unsuitability for dialysis due to coexisting conditions, including organ failure, long-term ventilator dependence, malnutrition, serious infectious diseases, terminal stage malignancy, or full dependence on caregivers [2,14,17,18,19]. In addition, in early 2019, Taiwan became the first country in Asia to officially implement a Patient Right to Autonomy Act promoting the concept of a good quality of death [12,13]. All individuals may establish an advance decision to decide what types of life-sustaining treatment they may refuse. With respect to the new patient autonomy law, clinicians were presented with the challenge of enhancing advance care planning for ESRD patients [5,13].

This study aimed to examine patient and health system characteristics and develop a patient assessment scale to evaluate end-stage renal disease (ESRD) patients for hospice care after implementation of the non-cancer hospice care reimbursement policy in 2009 in Taiwan. Specifically, this study used nationwide population-based inpatient administrative claims from the National Health Insurance Research Database and a registry of catastrophic illnesses to examine national trends and predictors of hospice care in Taiwan. The study findings may provide clinical implications for health professionals when engaging in advance care planning or shared decision making with ESRD patients.

## 2. Material and Methods

### 2.1. Study Design and Data Sources

We conducted a retrospective cohort study using nationwide population-based datasets in Taiwan, with 23 million population, including: (1) inpatient hospitalization discharge data, from which we obtained International Classification of Diseases, Ninth Revision, Clinical Modification (ICD-9-CM) diagnosis codes, dates of admission, and health provider characteristics; (2) a registry of catastrophic illnesses, from which we identified long-term dialysis patients and initiation dates. The two databases were linked using encrypted patient identifiers and all data analysis was completed in 2017–2018. This study was approved by the Institutional Review Board at Kaohsiung Medical University Hospital in Taiwan (KMUHIRB-EXEMPT(II)-20150081). All procedures performed in studies involving human participants were in accordance with the ethical standards of the institutional and/or national research committee and with the Helsinki declaration and its later amendments or comparable ethical standards. 

### 2.2. Study Population

**Figure 1:**
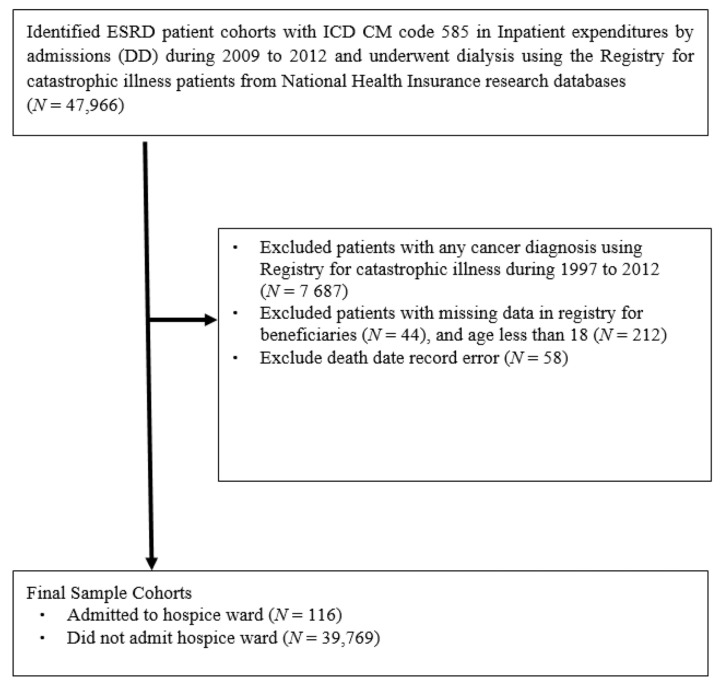
Study inclusion and exclusion criteria.

Using nationwide NHIRD claims data, we first included ESRD patients with a primary diagnosis code for CKD (ICD-9-CM code 585) for at least one inpatient hospitalization during the patient identification period, 1 January 2009 to 31 December 2012. We then used the registry of catastrophic illnesses to identify whether those patients received long-term dialysis before admission to hospice. We excluded patients aged younger than 18 years and those with any cancer diagnoses prior to hospice admission date. A total of 39,885 long-term non-cancer dialysis patients were included in the final analysis, and 116 of these received hospice care. Figure 1 shows the patient inclusion and exclusion criteria.

### 2.3. Variable Definitions

The major outcome of interest was that from an investigation of characteristics associated with the decision among non-cancer ESRD patients to receive hospice care. Based on literature and data availability, we included patient baseline demographic and clinical covariates and characteristics of the most frequently admitting hospitals. Demographic covariates included age, sex, income status (dependent, TWD 1–20,000, TWD 20,001–40,000, and TWD 40,000+), and residential area (urban, suburban, rural). Baseline clinical covariates included general comorbid conditions (Deyo-Charlson Comorbidity Index [CCI]), ESRD-related comorbid conditions (diabetes mellitus, hypertension, hyperlipidemia, heart failure, cerebrovascular disease, anemia, multiple organ dysfunction syndrome, sepsis). The CCI measures overall clinical condition by classifying or weighting comorbid conditions with consideration for both number and type of previously diagnose conditions, and has been widely used by researchers to indicate disease burden; low scores represent lower risks [20]. Frequency of baseline inpatient hospitalization was also measured as a proxy for physical condition and disease severity. 

In addition to patient characteristics, frequently admitting hospital characteristics may influence a patient or family’s decision, given the resources and capacities of individual health care institutions. To first address the issue of patients with multiple inpatient hospitalizations at different hospitals, we used a plurality provider algorithm to assign dialysis patients to their most frequently admitting hospitals, which we determined based on billing claims for the greatest number of admissions during the identification period [21]. Primary admitting hospital characteristics included accreditation level (medical center, regional hospital, and local hospital), ownership type (public, not-for-profit, for-profit), teaching hospital, presence of a hospice unit, and geographic location (Taipei, northern, central, southern, Kao-Ping, eastern). 

### 2.4. Statistical Analysis

We used multivariable logistic regression to estimate the likelihood of receiving hospice care among non-cancer dialysis patients. Given the concern that maximum likelihood estimates of the conventional logistic regressions may be biased by small numbers of events existing over a large sample size, the Firth penalized likelihood estimation model was used to reduce bias in generalized linear models [22]. Odds ratios (ORs) and 95% confidence intervals (CIs) are reported. We further used a receiver operating characteristic curve (ROC) analysis to determine the optimal models of a patient assessment index of hospice care in non-cancer dialysis patients. The estimated area under the ROC curve, and C-statistic were calculated. Youden’s index was used to find the optimal cut-off based on sensitivity and specificity results [23]. All statistical analyses were performed using SAS version 9.4 (SAS institute, Cary, NC) and Stata/SE 15.0. A *p* value < 0.05 was considered significant.

## 3. Results

Table 1 shows patient and frequently admitting hospital baseline characteristics for non-cancer ESRD patients who did and did not receive hospice care. Those who received hospice care tended to be older and sicker than those who did not. Patients who received hospice care were 59.48% male, mean age was 73.37 (±12.22) years (*p* < 0.001), 65.52% were dependent or low-income, 57.76% were resident in urban areas, mean CCI was 5.31 (*p* < 0.001), and 51.72% had diabetes mellitus, 72.41% hypertension (*p* < 0.003), 27.59% heart failure, 23.28% cerebrovascular disease (*p* < 0.001), 4.31% anemia (*p* < 0.001), 23.28% multiple organ dysfunction syndrome, 37.93% sepsis (*p* < 0.001), and 4.31% dementia (*p* = 0.02). In addition, these patients tended to have longer dialysis durations (mean, 4.92 years) (*p* = 0.002), and 82.76% had more than three hospitalizations (*p* < 0.001). 

Table 2 presents results from the multivariable logistic regression and Firth penalized likelihood models. Since the OR and 95% CI estimates from the two models were almost identical, results from the Firth penalized likelihood regression model are reported. Regarding other confounding factors, hospice care was more likely among older dialysis patients and those with comorbid anemia (OR 3.53, 95% CI 1.43–8.70), sepsis (OR 1.62, 95% CI 1.08–2.44), longer dialysis duration, more than 3 hospitalizations at baseline (OR 4.68, 95% CI 2.56–8.55), or a primary health provider with hospice units (OR 5.15, 95% CI 2.80–9.45). 

**Figure 2:**
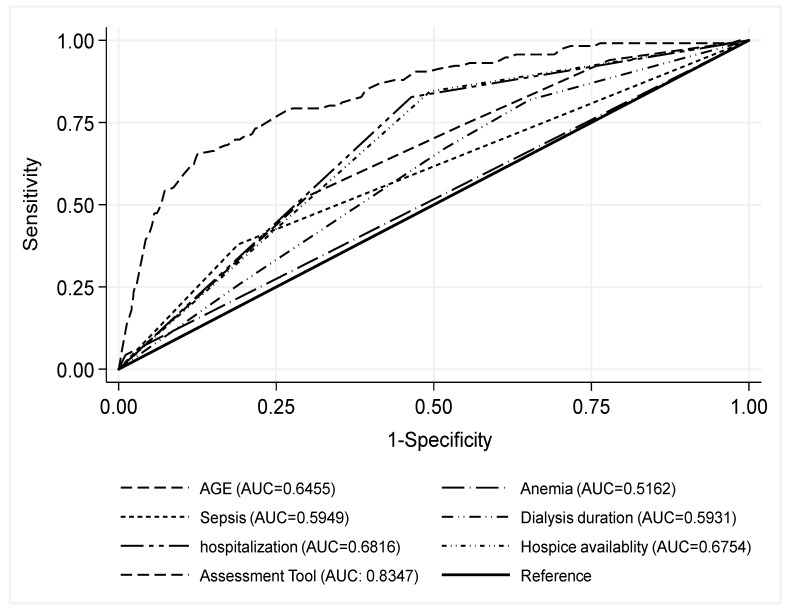
Receiver operating characteristic curve (ROC) for the key factors and patient assessment index for hospice care among non-cancer ESRD patients.

Given the full model results in Table 2, six key associated factors were selected and re-analyzed to develop a patient assessment of hospice care for dialysis patients (Table 3). Each factor was scored and weighted based on the odds ratio in the model, including age factors (age < 55 years, 0 points; 55–64 years, 3 points; 65–74 years, 3 points; >74 years, 6 points), anemia (no, 0 points; yes, 3 points), sepsis (no, 0 points; yes, 2 points), dialysis duration (<1 year, 0 point; 1–5 years, 3 points; 5–10 years, 5 points; >10 years, 5 points), counts of baseline hospitalizations (<3, 0 points; >3, 6 points), and primary hospital provider having hospice units (no, 0 points; yes, 6 points). The total score for the patient assessment tool was the sum of these six factors, ranging from 0 to 28. The area of the patient assessment index of hospice care under the ROC curve was 0.8347. Sensitivity and specificity were 0.6207 and 0.9007, respectively. Based on the Youden’s index, the suggested optimal cut-point for the patient assessment index of hospice care was 19 points, the positive predictive value (PPV) was 1.8%, and the negative predictive value (NPV) was 99.9%. Figure 2 shows the ROC curve for individual key factors and the patient assessment of hospice care.

## 4. Discussion

Despite hospice care being gradually recognized as a clinically appropriate treatment option for non-cancer ESRD patients, hospice services remain underutilized [8]. From the analysis in the current study, we identified a trend of extremely low hospice care utilization rates, even though increasing slightly from 0.03% in 2009 to 0.13% in 2012. Given the considerably higher intensity of treatment at the end of life for patients with ESRD, the issue of low access to hospice care among ESRD patients was also found in many countries when compared with other terminally ill patients [8,10,24]. Adequate capacity of institutions and health care workers, and supportive advance care planning may help to address patients’ end-of-life care needs. 

This study used nationwide population-based data to examine patient characteristics and primary health provider characteristics associated with hospice care for non-cancer dialysis patients in Taiwan after the nationwide non-cancer hospice care reimbursement policy implemented in 2009. Regarding patient demographic and clinical characteristics, consistent with previous studies [14,25], this current study found that older dialysis patients were more likely to receive hospice care, especially those older than 75 years. In addition, previous studies showed that functional status may be poor at the time of dialysis withdrawal [25,26,27,28]. The current study found that dialysis patients with specific dialysis-related complications (sepsis or anemia) were more likely to withdraw from dialysis and receive hospice care. Sepsis is the most frequent cause of acute kidney injury, and sepsis or septic shock are common in dialysis patients [29,30]. Patients may have poor renal outcomes and greater death risk with sepsis and septic shock [29,30,31,32]. Samak and Jaber (2000) compared annual mortality rates caused by sepsis in patients with ESRD in the United States and found dialysis patients to have approximately 100- to 300-fold higher mortality risk due to septicemia compared with the general population [33]. Anemia is also a common complication of uremia and a major contributor to morbidity and mortality in ESRD patients [34]. One recent systematic review by Palaka et al. (2021) searched literature published from 2002 to 2018 and found that hemoglobin level or anemia in CKD patients on dialysis were associated with greater all-cause mortality [34]. In addition, ESRD patients with longer dialysis durations and more frequent hospitalizations were more likely to receive hospice care given that health-related quality of life may decline as dialysis duration increased [8]. 

With respect to health system factors, this study found that ESRD patients were more likely to receive hospice care when their frequently admitting hospitals had hospice units. This may be because the accessibility of health service hospice care differed across countries, thus affecting patient or family decisions regarding hospice care. One recent study compared hospice systems in the United States, Taiwan, and Japan, and found significant differences across countries [35]. In the United States, most hospice providers were free-standing home hospice institutions, whereas hospitals provided hospital-based inpatient and outpatient hospice care and home hospice services in Taiwan. As of year 2021, 82 inpatient palliative care units, 160 inpatient consultation teams or hospice-shared care teams, and 125 home-care teams were available in Taiwan [35,36,37].

Given low quality of life and increased dependency on others among ESRD patients with long-term continuous dialysis, it is important to develop a hospice care screening tool to evaluate all ESRD patients regularly for health professionals to launch advance care planning (ACP) processes [6,13,38]. Existing predictive models were based on predictive prognosis or mortality [25,26,27]. For example, Robins and Katz (2013) reviewed and documented five key variables to determine risk of death or prognosis and considered non-dialysis pathways for ESRD patients, including nephrologist responses to the surprise question relating to the potential for dying in the next 6 to 12 months among patients who are elderly; have significant comorbidity, poor functional status (e.g., peripheral vascular disease, dementia, poor serum albumin level), or malnutrition; or who reside in a nursing home [25,26,27,28]. While the importance of early integration of palliative care among terminally ill non-cancer patients has been addressed in recent studies [24,39], initiations of conversations were often challenging for nephrologists when engaging in ACP discussion with their patients. Through this study, a useful tool with predictive factors for hospice care among non-cancer ESRD patients could be used for making care plans in clinical practices.

The current study used Taiwanese national population-based data to identify ESRD patients who may use hospice care and to develop a patient assessment of hospice care as a screening tool. This tool consisted of six key associated factors, including age factors, anemia, sepsis, dialysis duration, counts of baseline hospitalizations, and primary hospital provider having hospice units. By means of retrospective data analysis from real-world databases, our study further used a six-item patient assessment index of hospice use based on the likelihood of hospice use in non-cancer ESRD patients. Total scores may range from 0 to 28, with an optimal cut-off of 19. The positive predictive value (PPV) was 1.8%, indicating that of those with scores higher than 19 points, 1.8% received hospice care. The negative predictive value (NPV) was 99.9%, indicating that of those with scores lower than 19 points, 99.9% did not receive hospice care. Due to the low prevalence of hospice use in the ESRD population, the sensitivity and PPV value were small, but still approximately 13.85 times greater than the prevalence rate of hospice use in 2012 [23]. Based on this tool, health professionals may efficiently target patients potentially or highly willing to receive hospice care and engage in shared decision making and advance care planning with ESRD patients and family members [5,6,7,14].

Our study primarily used secondary data from inpatient administrative insurance claims and a registry of catastrophic illnesses to study the research questions. Several advantages included: a large population sample, saving time and money required to perform patient assessments of hospice care, and longitudinal data to detect prevalence of and factors associated with hospice care in non-cancer ESRD patients. However, this study also has limitations. First, over- or under-diagnosis may occur because the conditions of interest were defined using ICD-9-CM codes in administrative claims. Second, several unobserved confounders regarding individual patients (e.g., functional status, mental status, and nutritional status) were not available. Third, we identified only hospital-based hospice care due to limited data availability. ESRD patients may receive palliative care through outpatient follow-up or home-care hospice care. Finally, the current study was conducted on a single population in Taiwan, so the results may not generalize to other countries.

## 5. Conclusions

In conclusion, the current study identified factors associated with hospice care and developed a proper patient assessment scale of hospice care for non-cancer dialysis patients in Taiwan. This screening tool may help health professionals efficiently target patients and family members potentially or highly willing to receive hospice care and engage in shared decision making or advance care planning at the end of life with ESRD patients.

## Figures and Tables

**Table 1:** Comparisons of patient and healthcare provider characteristics between ESRD patients who did and did not receive hospice care in Taiwan.

	Received Hospice Care	Did Not Receive Hospice Care	
	Mean ± SD/(*N*, %)	Mean ± SD/(*N*, %)	*p*-value
*N*	116	39,769	
Patient demographic characteristics			
Sex			
Male (*N*, %)	69 (59.48%)	20,617 (51.84%)	0.100
Female (*N*, %)	47 (40.52%)	19,152 (48.16%)	
Age on index date (in years, mean ± SD)	73.37 (±12.22)	65.33 (±14.24)	<0.001
Age categories (*N*, %)			
<55	7 (6.03%)	8828 (22.20%)	<0.001
55–64	23 (19.83%)	9291 (23.36%)	
65–74	25 (21.55%)	9729 (24.46%)	
≥75	61 (52.59%)	11,921 (29.98%)	
Income categories (*N*, %)			
Dependent	28 (24.14%)	9873 (24.83%)	0.548
1–20,000	48 (41.38%)	14,281 (35.91%)	
20,001–39,999	37 (31.90%)	13,881 (34.90%)	
≥40,000	3 (2.59%)	1734 (4.36%)	
Residence area			
Urban area	67 (57.76%)	20,978 (52.75%)	0.548
Suburban area	35 (30.17%)	13,671 (34.38%)	
Rural area	14 (12.07%)	5120 (12.87%)	
Patient clinical characteristics			
CCI (mean ± SD )	5.31 (±3.37)	3.16 (±2.31)	<0.001
CCI categories (*N*, %)			
0	6 (5.17%)	7323 (18.41%)	<0.001
1–2	14 (12.07%)	9276 (23.32%)	
≥3	96 (82.76%)	23,170 (58.26%)	
Clinical comorbid conditions (*N*, %)			
Diabetes mellitus	60 (51.72%)	17,838 (44.85%)	0.137
Hypertension	84 (72.41%)	23,366 (58.75%)	0.003
Hyperlipidemia	4 (3.45%)	2755 (6.93%)	0.140
Heart failure	32 (27.59%)	9619 (24.19%)	0.393
Cerebrovascular disease	27 (23.28%)	5135 (12.91%)	<0.001
Dialysis complications (*N*, %)			
Anemia	5 (4.31%)	426 (1.07%)	<0.001
Multiple organ dysfunction syndrome	27 (23.28%)	9511 (23.92%)	0.872
Sepsis	44 (37.93%)	7536 (18.95%)	<0.001
Other disease risk			
Dementia (*N*, %)	5 (4.31%)	526 (1.32%)	0.020
Dialysis duration (in years, mean ± SD )	4.92 (±4.22)	4.16 (±4.51)	0.070
Dialysis duration categories (*N*, %)			
Within 1 year	21 (18.10%)	13,846 (34.82%)	0.002
1 year to 5 years	48 (41.38%)	12,987 (32.66%)	
5 years to 10 years	30 (25.86%)	7623 (19.17%)	
More than 10 years	17 (14.66%)	5313 (13.36%)	
Counts of hospitalization (within one year prior to index date)	6.05 (±4.08)	3.02 (±2.90)	<0.001
Categories of hospitalization within one year prior to index date (*N*, %)			
<3 times	20 (17.24%)	21,303 (53.57%)	<0.001
≥3 times	96 (82.76%)	18,466 (46.43%)	
Primary hospital provider characteristics			
Health institution accreditation level (*N*,%)			
Medical center	54 (46.55%)	14,218 (35.75%)	<0.001
Regional hospital	57 (49.14%)	18,710 (47.05%)	
Local hospital	5 (4.31%)	6841 (17.20%)	
Ownership type (*N*, %)			
Public	21 (18.10%)	12,269 (30.85%)	<0.001
Not-for-profit	89 (76.72%)	18,766 (47.19%)	
For-profit	6 (5.17%)	8734 (21.96%)	
Teaching hospital (*N*, %)			
Yes	111 (95.69%)	34,838 (87.60%)	0.005
No	5 (4.31%)	4931 (12.40%)	
Having hospice unit (*N*, %)			
Yes	98 (84.48%)	19,650 (49.41%)	<0.001
No	18 (15.52%)	20,119 (50.59%)	
Location of health care institution (*N*, %)			
Taipei	41 (35.34%)	11,748 (29.54%)	<0.001
Northern	14 (12.07%)	7241 (18.21%)	
Central	18 (15.52%)	8409 (21.14%)	
Southern	17 (14.66%)	3887 (9.77%)	
Kao-Ping	9 (7.76%)	7105 (17.87%)	
Eastern	17 (14.66%)	1379 (3.47%)	

CCI = Charlson comorbidity index; ESRD = end-stage renal disease.

**Table 2:** Logistic regression model and Firth penalized likelihood model results regarding the associated factors related to hospice care among ESRD patients in Taiwan.

	Logistic Regression			Firth Penalized Likelihood		
	OR	95% CI	*p*-value	OR	95% CI	*p*-value
Patient demographic characteristics						
Sex (Ref.: female)						
Male	1.24	(0.85, 1.83)	0.267	1.24	(0.84, 1.82)	0.274
Age (Ref.: <55)						
55–64	3.27	(1.39, 7.70)	0.007	3.11	(1.35, 7.14)	0.008
65–74	3.15	(1.33, 7.47)	0.009	2.99	(1.29, 6.93)	0.010
≧75	6.49	(2.88, 14.61)	0.000	6.07	(2.76, 13.34)	0.000
Income categories (Ref.: dependent)						
1–20,000	0.95	(0.58, 1.55)	0.837	0.94	(0.58, 1.53)	0.816
20,001–39,999	0.94	(0.56, 1.58)	0.809	0.93	(0.56, 1.57)	0.796
≧40,000	1.05	(0.31, 3.55)	0.939	1.19	(0.38, 3.71)	0.766
Place of residence (Ref.: urban)						
Suburban	0.85	(0.54, 1.33)	0.475	0.86	(0.55, 1.33)	0.493
Rural	0.72	(0.38, 1.36)	0.313	0.74	(0.39, 1.39)	0.345
Patient clinical characteristics						
CCI categories (Ref.: 0)						
1–2	1.13	(0.41, 3.12)	0.812	1.12	(0.42, 2.99)	0.823
≧3	2.27	(0.84, 6.13)	0.105	2.19	(0.83, 5.75)	0.112
Clinical comorbid conditions (Ref.: No)						
Diabetes mellitus	0.78	(0.51, 1.19)	0.253	0.78	(0.51, 1.19)	0.249
Hypertension	0.95	(0.59, 1.53)	0.841	0.95	(0.59, 1.52)	0.815
Hyperlipidemia	0.38	(0.14, 1.05)	0.062	0.43	(0.16, 1.11)	0.082
Heart failure	0.76	(0.49, 1.17)	0.211	0.76	(0.50, 1.18)	0.224
Cerebrovascular disease	1.25	(0.79, 1.97)	0.337	1.26	(0.80, 1.98)	0.312
Dialysis complications (Ref.: No)						
Anemia	3.28	(1.27, 8.42)	0.014	3.53	(1.43, 8.70)	0.006
Sepsis	1.62	(1.08, 2.45)	0.020	1.62	(1.08, 2.44)	0.020
Multiple organ dysfunction syndrome	0.62	(0.38, 1.00)	0.051	0.62	(0.38, 1.01)	0.055
Other disease risks (Ref.: No)						
Dementia	1.50	(0.59, 3.80)	0.396	1.62	(0.67, 3.95)	0.286
Dialysis duration (Ref.: <1 year)						
1 year to 5 years	2.80	(1.63, 4.79)	0.000	2.75	(1.62, 4.69)	0.000
5 years to 10 years	3.58	(1.95, 6.57)	0.000	3.54	(1.94, 6.46)	0.000
More than 10 years	3.74	(1.84, 7.58)	0.000	3.73	(1.85, 7.51)	0.000
Counts of hospitalization within one year prior (Ref: < 3 times)						
≧ 3 times	4.79	(2.60, 8.80)	0.000	4.68	(2.56, 8.55)	0.000
Primary hospital provider characteristics						
Health care institution accreditation level (Ref.: local hospital)						
Medical center	3.00	(0.67, 13.44)	0.152	2.75	(0.60, 12.67)	0.193
Regional hospital	2.18	(0.51, 9.39)	0.296	2.03	(0.46, 8.97)	0.353
Ownership type (Ref.: For-profit)						
Public	2.05	(0.77, 5.45)	0.152	1.96	(0.76, 5.09)	0.166
Not-for-profit	0.63	(0.21, 1.88)	0.407	0.62	(0.21, 1.79)	0.377
Teaching hospital (Ref.: no)						
Yes	0.51	(0.12, 2.07)	0.344	0.49	(0.12, 2.04)	0.324
Having hospice units (Ref.: no)						
Yes	5.37	(2.90, 9.94)	0.000	5.15	(2.80, 9.45)	0.000
Location of health care institution (Ref.: Taipei area)						
Northern area	1.65	(0.85, 3.23)	0.139	1.69	(0.87, 3.25)	0.119
Central area	0.70	(0.37, 1.33)	0.278	0.72	(0.39, 1.35)	0.307
Southern area	1.19	(0.64, 2.19)	0.584	1.22	(0.66, 2.23)	0.525
Kao-Ping area	0.42	(0.20, 0.90)	0.026	0.44	(0.21, 0.94)	0.033
Eastern area	3.06	(1.62, 5.79)	0.001	3.10	(1.66, 5.81)	0.000

CCI = Charlson comorbidity index.

**Table 3:** Patient assessment index for hospice care among ESRD patients in Taiwan.

Key Factors	OR	95% CI	*p*-Value	Scoring
Primary patient characteristics				
Age categories				
<55 (ref.)	-	-	-	0
55–64	3.00	(1.28, 7.02)	0.011	3
65–74	3.03	(1.31, 7.05)	0.010	3
≧75	6.31	(2.86, 13.91)	0.000	6
Anemia				
No (ref.)	-	-	-	0
Yes	2.94	(1.17, 7.36)	0.021	3
Sepsis				
No (ref.)	-	-	-	0
Yes	1.62	(1.10, 2.41)	0.016	2
Dialysis duration				
< 1 year (ref.)	-	-	-	0
1 year to 5 years	3.38	(2.02, 5.67)	0.000	3
5 years to 10 years	4.98	(2.81, 8.80)	0.000	5
More than 10 years	5.35	(2.76, 10.37)	0.000	5
Counts of hospitalization within one year prior				
<2 (ref.)	-	-	-	0
≧3 times	5.62	(3.38, 9.35)	0.000	6
Primary hospital providers’ characteristics				
Having hospice units				
No (ref.)	-	-	-	0
Yes	5.91	(3.56, 9.79)	0.000	6

Total Score Range (Min, Max) = (0,28); Area Under ROC curve (AUC)/ C-Statistics = 0.8347 (95%CI = 0.7972, 0.8722); Optimal Cut-Point Score = 19; Optimal Sensitivity = 0.6207; Optimal Specificity = 0.9007; Positive predictive value (PPV) = (68/3676) = 1.8%; Negative predictive value (NPV) = (35,819/35,863) = 99.9%.

## Data Availability

Restrictions apply to the availability of these data. Data was obtained from the National Health Insurance Research Database and are available with the permission.
